# PepGen: conditional generation of peptides for MHC binding

**DOI:** 10.1093/bioinformatics/btag464

**Published:** 2026-08-21

**Authors:** Dani Korpela, Alexandru Dumitrescu, Martin Stražar, Rui Li, Ramnik J Xavier, Daniel B Graham, Harri Lähdesmäki

**Affiliations:** Department of Computer Science, Aalto University, Espoo, 02150, Finland; Broad Institute of MIT and Harvard, Cambridge, MA, 02142, United States; Department of Computer Science, Aalto University, Espoo, 02150, Finland; Broad Institute of MIT and Harvard, Cambridge, MA, 02142, United States; Broad Institute of MIT and Harvard, Cambridge, MA, 02142, United States; Department of Biotechnology and Systems Biology, National Institute of Biology, Ljubljana, 1000, Slovenia; Broad Institute of MIT and Harvard, Cambridge, MA, 02142, United States; Broad Institute of MIT and Harvard, Cambridge, MA, 02142, United States; Broad Institute of MIT and Harvard, Cambridge, MA, 02142, United States; Department of Computer Science, Aalto University, Espoo, 02150, Finland

## Abstract

**Motivation:**

Peptide-MHC II binding drives adaptive immunity, yet discovery of novel binder peptides remains challenging due to open binding grooves of MHC-II that accommodate variable-length peptides. While discriminative models perform well, they are unfeasible for generation via enumeration due to vast peptide space ($20^{13}\approx 8\times 10^{16}$ for peptides of length 13 amino acids). Generative AI approaches could accelerate binder design to enable vaccines targeted to particular MHC-II alleles or optimize other peptide chemical properties.

**Results:**

We introduce PepGen, the first protein language model for MHC II peptide generation building on Generalized Language Modeling. PepGen conditions on alleles, arbitrary partial peptides including putative TCR-interacting motifs, and continuous binding affinity. Across multiple benchmarks including infilling and *de novo* generation, PepGen outperformed frequency sampling, Gibbs clustering, and autoregressive baselines. Adjusted log-probabilities enable good classification performance. Experimental validation confirmed that the SARS-CoV-2 peptide TEGALNTPKDHIGTR binding the HLA-DQA101:03-DQB106:03 allele can be redesigned to bind the HLA-DQA101:02-DQB105:02 allele. PepGen generated three putative TCR-motif-preserving binders gaining up to 70% of original MFI. Overall, PepGen provides scalable, motif-constrained MHC II peptide redesign and *de novo* generation, validated through thorough benchmarks and functional assays.

**Availability and implementation:**

Code and Data are available at https://github.com/DaniTheOrange/PepGen.

## 1 Introduction

The T cell based adaptive immune system is crucial in defending the body against pathogens and cancer. For an immune reaction to take place, the T cell receptor (TCR) has to bind to an epitope (peptide) presented by the MHC molecule. Consequently, peptide–MHC binding is a prerequisite for TCR recognition and activation of the immune response.

The MHC genes are the most polymorphic in the human genome, and the repertoire of peptides that can bind to a given MHC molecule is determined by its amino acid sequence. The MHC is divided into two canonical classes: MHC class I, which contains a closed binding groove and typically presents peptides of length 8–10 amino acids, and MHC class II, which features an open binding groove capable of binding longer peptides. In MHC class II, a 9-mer core fits within the groove with flanking residues extending outward; anchor positions P1, P4, P6, and P9 within this core provide the primary MHC contacts driving binding specificity. The open binding groove and core-flanking regions, together with the heterodimeric structure, make the peptide–MHC class II (pMHC II) interaction considerably more complex.

Numerous computational models have been developed for predicting peptide–MHC binding, achieving excellent performance for class I and good performance for class II (You *et al*. 2022, Nilsson *et al*. 2023). Beyond discriminative modeling, generative modeling offers an alternative paradigm: rather than classifying peptides as binders or non-binders, generative models aim to learn and reproduce the underlying distribution of binding peptides for specific MHC alleles.

Generative peptide models have been developed for MHC class I (Chen *et al*. 2023), but no prior generative models exist for the more challenging MHC class II. Given the exponential scaling of *n*-length peptides, Dai *et al*. (2021) explored synthetic pMHC II design in a restricted manner, enumerating only the four anchor positions (P1, P4, P6, P9), followed by discriminative filtering; however, peptide regions beyond these anchors—including core-flanking residues—also significantly influence binding affinity, as reflected in recent classifiers like DeepMHCII (You *et al*. 2022). Conversely, generative modeling enables the tractable design of an unlimited number of residues.

TCR binding motifs, i.e. patterns of amino acids recognized by specific TCRs, offer additional design constraints for generating immunogenic pMHC II complexes. These motifs, often comprising non-anchor core residues (though not always all positions), can be feasibly identified through alanine scanning mutagenesis studies (Yang *et al*. 2014, Ishii *et al*. 2023). Incorporating such motifs could enable targeted generation of peptides that are both MHC-compatible and TCR-recognizable.

Designing peptides through infilling of larger segments (e.g. all but TCR motif amino acids) or generating entirely novel sequences *de novo* presents great challenges, as the combinatorial space scales prohibitively with length: a 13-mer peptide comprises approximately $8.2\times 10^{16}$ possible sequences. Exhaustive *in silico* or *in vitro* exploration is, therefore, infeasible. A generative model that biases sampling from random sequences toward likely MHC II binders could substantially accelerate discovery, serving as the first step in a pipeline of computational screening and experimental validation.

**Figure 1:**
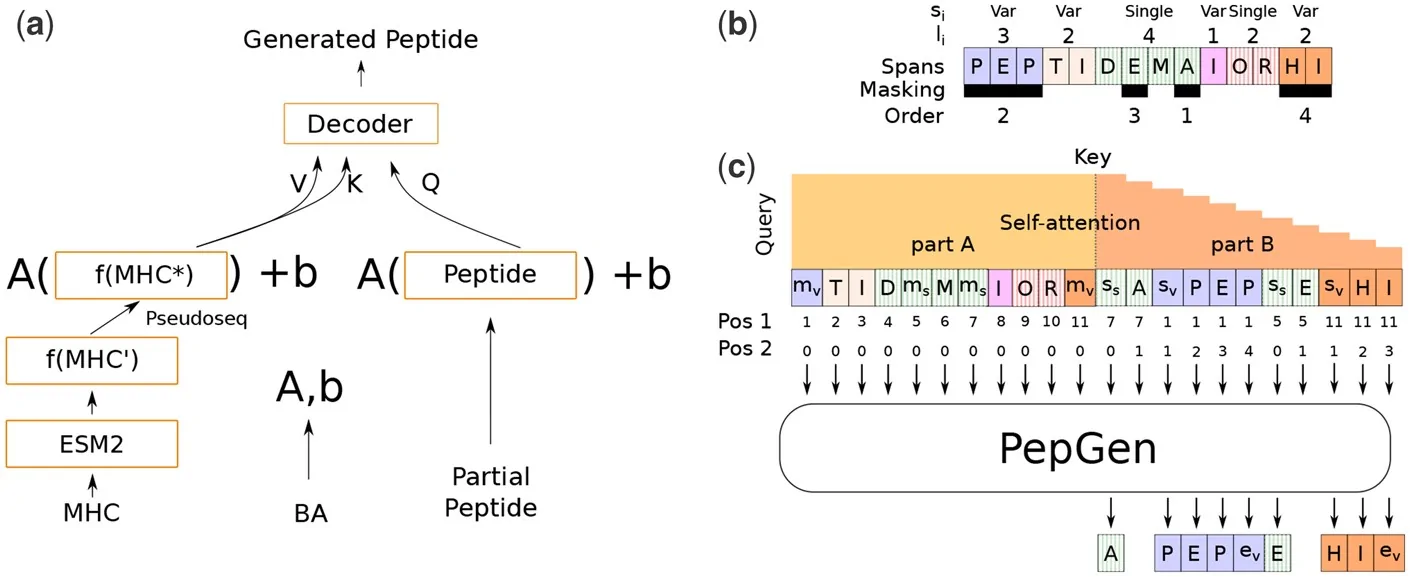
(a) Illustration of the PepGen model. The model takes the ESM2 embedded pseudosequence of the MHC, the partial peptide and the target BA as input. When starting from an empty sequence the partial peptide conditioning consists only of masking token(s). (b, c) Illustration of the GLM objective. (b) First the training peptide is divided into spans of which some are sampled to be masked. Then the generation order is sampled. Examples of spans with their types ($s_{i}$), lengths ($l_{i}$), masking, and order (o) are shown. (c) According to the sampled masking configuration, the model predicts auto-regressively the masked amino acids. Input is shown above the model and target below. The generation allows full attention to the partial peptide and a causal self-attention mask is applied for the infilled/generated part. Here $m_{v}$, $m_{s}$, $s_{v}$, $s_{s}$, and $e_{v}$ denote the variable mask, single mask, variable start of sequence, single start of sequence, and variable end of sequence, respectively.

In this work, we present *PepGen*, a protein language model based generative framework for MHC class II peptide generation. To our knowledge, PepGen is the first fully generative model specifically designed for this task. Our model may be conditioned on partial peptide information, i.e. arbitrary subsets of amino acids considered fixed in the final synthetic peptide (e.g. with TCR motifs fixed and variable length segments generated via infilling), MHC II binding groove residues, and binding affinity (BA) targets (Fig. 1a).

We benchmark PepGen against established generative and discriminative baselines, analyze the impact of affinity conditioning, and introduce a parameter scheduling strategy that balances generation quality and diversity. Finally, we validate PepGen generated sequences using peptide-MHC binding assays, demonstrating its ability to generate MHC II binders and establishing its utility as the first step in a discovery pipeline combining computational design, putative TCR motif incorporation, screening, and experimental validation.

## 2 Materials and methods

### 2.1 PepGen

*PepGen* employs an adapted generalized language modeling (GLM) objective (Du *et al*. 2022) that unifies masked language modeling with autoregressive generation, enabling conditional synthesis of variable-length MHC class II peptides. We modify the original autoregressive blank infilling formulation from GLM with higher masking ratios and by adding *single spans* with a separate Poisson masking distribution. We enable peptide generation conditioned on arbitrary amino acid subsets by interleaving special mask tokens with residue subsequences. This defines the *partial peptide* (or *Part A* in the GLM formulation) provided as model input. Each mask token is associated with a corresponding start token that prompts the model to generate the missing residues at that position (denoted *Part B*). During training, start tokens are randomly shuffled to expose the model to all possible generation orders. Furthermore, by employing single and variable mask token types, the framework allows flexible control over whether each mask is replaced by one or multiple (length inferred by the model) amino acids. The resulting adapted GLM model, which emphasizes generative capability over simple infilling, is described below.

#### 2.1.1 GLM training objective

A peptide is a sequence $x=(x_{1},\ldots ,x_{L})$, where each $x_{i}\in \{A,C,D,\ldots \}$ is an amino acid token. Each datapoint comprises of {x,MHC,y} where *y* is either continuous affinity value for BA data or a constant for eluted ligand (EL) data and MHC encodes an MHC sequence.

A *span* of a peptide sequence is defined as a consecutive subsequence of amino acid tokens. In a *variable span* either all or none of the tokens are masked, whereas in a *single span* each amino acid is masked independently. An extended token vocabulary is denoted as $V=\{A,C,D,\ldots \}\cup \{m_{v},s_{v},e_{v},m_{s},s_{s}\}$, where the masking tokens hide all amino acids ($m_{v}$) or a single amino acid ($m_{s}$) in variable or single spans, respectively, $s_{v}$ and $e_{v}$ denote the start and end of a variable span, and $s_{s}$ is the start of a single span.

For each training peptide sequence x, at every epoch, we sample a masking configuration c which randomly divides the sequence into $N_{s}$ spans defined by their span types $s_{i}\in \{\text{single},\text{variable}\}$ and span lengths $l_{i}\in Z_{+}$, for $i\in \{1,\ldots ,N_{s}\}$ (Fig. 1b). When $s_{i}=\text{single}$, each residue within the span has an associated binary variable and is replaced with $m_{s}$ when the variable is 1. When $s_{i}=\text{variable}$, a single binary variable masks the entire subset of amino acids (all tokens are replaced by one $m_{v}$). Thus, masking is formally defined by $m_{i}\in {\left\{0,1\right\}}^{1+1_{\text{single}}(s_{i})(l_{i}-1)}$, where 1 is an indicator function. Lastly, the generation order is specified by $o\in S_{N_{m}}$, where $S_{N}$ is the set of all permutations with *N* elements, and $N_{m}=\sum_{i=1}^{N_{s}}\sum_{j=1}^{l_{i}}m_{j}$ is the total number of single and variable masks. Collectively, the masking configuration is formed by $c=\{{\left\{s_{i},l_{i},m_{i}\right\}}_{i=1}^{N_{s}},o\}$. We use shifted (by 1) and truncated Poisson distributions to disallow spans of length 0 and to keep the spans within the sequence length such that $\sum_{i=1}^{N_{s}}l_{i}=L$. The masking configuration c is sampled using the following steps:

1. Sample span type: $s_{i}∼$ Bernoulli($P_{\text{span}\_\text{type}}$).
2. If $s_{i}=\text{variable}\;\text{span}$
3.  Sample span length: $l_{i}∼\text{Po}(\lambda_{\text{var}})+1$
4.  Replace all tokens with one $m_{v}$ with Bernoulli($P_{\text{var}}$).
5. Else ($s_{i}=\text{single}\;\text{span}$)
6.  Sample span length: $l_{i}∼\text{Po}(\lambda_{\text{single}})+1$.
7.  Replace each token independently with $m_{s}$ with Bernoulli($P_{\text{single}}$).
8. Repeat until sequence end.
9. Sample mask generation order.

A detailed pseudocode for the sampling process is given in Appendix Algorithm 1, available as supplementary data at *Bioinformatics* online. Hyperparameters $P_{\text{span}}$, $P_{\text{var}}$, $P_{\text{single}}$, $\lambda_{\text{var}}$ and $\lambda_{\text{single}}$ control the sampling process.

Given a configuration c, the model input (Part A), is formed by corrupting the original x with masks according to ${\left\{m_{i}\right\}}_{i=1}^{N_{s}}$. Similarly, the output (Part B) is formed by concatenating $s_{s}$ with the amino acids masked by each $m_{s}$, and $s_{v}$ with the subsets of amino acids masked by each $m_{v}$ plus $e_{v}$, and reordering those according to the configuration’s sampled generation order o (see Fig. 1c for illustration). The training objective is to reconstruct x conditioned on its fully or partially masked version, MHC, and *y* (conditioning details in Section 2.1.3):


(1) $$P_{\theta }(“\text{Part}\;B”|“\text{Part}\;A”,\text{MHC},y),$$

where we use a 4-layer transformer decoder as the prediction head with a causal mask for Part B and train the model using the cross-entropy loss (Fig. 1c). That is, the neural network reconstructs the masked-out tokens (i.e. Part B), given the partial peptide (i.e. Part A), the MHC, and the BA or EL value *y*.

#### 2.1.2 Probability of a sequence

A masking configuration c for x depends only on the sequence length *L*, not on the amino acids x, and can be computed as:


(2) $$\begin{array}{c} P(c|L)=P_{\text{span},\text{type}}\cdot P_{\text{var},\text{len}}\cdot P_{\text{single},\text{len}}\cdot P_{\text{last}}\cdot \\ P_{\text{var},\text{mask}}\cdot P_{\text{single},\text{mask}}\cdot P_{\text{order}}. \end{array}$$

A detailed description of each factor can be found in Appendix Section 3, available as supplementary data at *Bioinformatics* online. The probability of a sequence can be evaluated by the model given that Part B is the original peptide (with sampled generation order) and, correspondingly, Part A comprises only of masking tokens. By requiring $P_{\text{var}}=P_{\text{single}}=1$ the probability of the sequence in one sampled generation order can be evaluated $P_{\theta }(“\text{Part}\;B”|“\text{Part}\;A”,\text{MHC},y,P_{\text{var}}\;=\;P_{\text{single}}\;=\;1)$, which is equivalent to $P_{\theta }(x|c,\text{MHC},y,P_{\text{var}}=P_{\text{single}\;}=1)$. For classification purposes, we approximate the sequence probability using Monte Carlo as


$$P(x|\text{MHC},y)\approx \frac{1}{N}\sum_{i=1}^{N}P_{\theta }(x|c_{i},\text{MHC},y,P_{\text{var}}=P_{\text{single}}=1),$$

where $c_{i}∼P(\cdot |L)$ are obtained using the sampling process from Section 2.1.1. As the sequence probability of an autoregressive model is the product of the individual tokens leading to smaller probabilities for longer sequences, we use the geometric average of the token probabilities, i.e. $P_{\text{adj}}(x)=\prod_{i=1}^{L}P{\left(x_{i}\right)}^{1/L}$ as the classification score. This corresponds to the arithmetic average of token log probabilities: $\frac{1}{L}\sum_{i=1}^{L}\;\text{log}\;\;P(x_{i})$.

#### 2.1.3 Model architecture and conditionings

*PepGen* uses a 4-layer transformer decoder that autoregressively infills masked peptide sequences conditioned on partial peptide (i.e. Part A), MHC, and BA or EL target value *y* as in Equation (1) (Fig. 1a). The masked peptide uses one-hot encoding with special tokens for variable- and single-token masks. Fully masked inputs enable *de novo* generation. The conditional binding affinity is a scalar value obtained following (Nilsson *et al*. 2023) by transforming the experimental binding affinities with 1−log(aff)/ log(50 000) and capping the value between 0 and 1. EL data receives a constant value −1. The MHC conditioning is done by embedding the full sequence with ESM2-650M, then subsampling representations of 34 peptide-contacting residues from the binding groove. The MHC conditioning is used as the keys in cross-attention while the scalar *y* either BA ([0,1]) or EL (−1) conditioning applies an affine transform (shift+scale) to both masked peptide and MHC representations. The partial peptide is given as amino acid sequence with masking tokens that is converted into one-hot representation for the model. We retain the 2-D positional encodings from the original GLM, which is used to relate the generated tokens to the masks and extended it for single-token masks. Hyperparameters used can be found in Appendix Section 2, available as supplementary data at *Bioinformatics* online.

#### 2.1.4 Post-training generation adjustments

Generation uses nucleus sampling (P∈[0,1]) (Holtzman *et al*. 2020) and classifier-free guidance (CFG) adapted for autoregressive models (Sanchez *et al*. 2024, Njirjak *et al*. 2024). For CFG, models were trained with 5% unconditional samples (*null* MHC/BA-EL conditioning). During inference, conditioned on MHC allele and BA ([0,1]) or EL (−1), guidance is applied in the token logit space before softmax:


$$\ell_{\text{CFG}}=\ell_{\text{uncond}}+s\cdot (\ell_{\text{cond}}-\ell_{\;\text{uncond}}),$$

where $\ell_{\text{cond}}$ and $\ell_{\text{uncond}}$ are conditional and unconditional model logits, respectively, and s≥1 amplifies the conditional signal.

To adapt (s,p) parameters to each conditioning context (allele, BA, partial peptide), we implemented adaptive scheduling. The scheduler starts from one of three initializations: (7,1), (0,5.5), or (1,0.3); then progressively steps toward the neutral point (s=1, p=1) based on sequence novelty.

After each batch, if the fraction of unique sequences falls below 0.77, the scheduler takes a constant-length step toward (1,1). Steps are sized according to batch size and total samples, ensuring the neutral point is reached by the halfway mark (worst case). We used all three starting points by generating one-third of samples from each, unless otherwise specified. For further details on post-training parameter ablation see Appendix Section 1, available as supplementary data at *Bioinformatics* online.

### 2.2 Data

We constructed two cross-validation folds ($D_{0}$, $D_{1}$) from the Nilsson *et al*. (2023) dataset, which comprises single-allelic and multi-allelic mass spectrometry EL data alongside binding affinity (BA) data. EL data contains experimentally observed presented peptides (positive examples only) augmented with artificial negatives, while BA data provides continuous binding measurements. We use only the single-allelic EL data and discard the artificial negatives totaling in 172 880 datapoints across 57 alleles while BA data contains 127 749 for 73 alleles. Each fold $D_{i}$ consists of training ($D_{i,\text{train}}$) and validation ($D_{i,\text{val}}$) subsets, and the two folds are themselves separated by strict 9-mer separation: no peptide shares any 9-mer subsequence across MHC alleles between $D_{0}$ and $D_{1}$, ensuring biologically meaningful splits that prevent sequence leakage while preserving allele distributions. Most experiments used binding affinity (BA) data only, excluding EL. Generative models are trained on $D_{i}$ while test DeepMHCII discriminators on held-out $D_{1-i}$, maintaining similar-yet-distinct pMHC distributions.

Based on the validation subset of the test fold ($D_{1-i,\text{val}}$), we constructed a conditional generation benchmark, denoted as $D_{\text{bot}}^{\left(\text{gen}\right)}$. We selected 37 alleles for this benchmark, based on sufficient availability of training data. For each allele, we further selected the two lowest-affinity (high nM) pMHC pairs (if available) from the test-validation split. As no experimental structural data were available for these peptides, we used the DeepMHCII discriminator trained on the same fold as the generative model ($D_{i}$) to predict the peptide binding cores. Canonical anchor positions and residues outside the predicted core were masked, leaving only the non-anchor core residues, which are presumed to primarily drive T cell receptor (TCR) binding. We refer to these residues as the *putative TCR motif*. The conditional generation task was thus defined as generating peptide binders that preserve the inferred putative TCR motif.

For further evaluation, we used the EL dataset from (Stražar *et al*. 2023) (CAPTAn), which includes both positive samples and artificial negatives. For generative performance evaluation, we sampled 100 positive pMHC pairs for the same 37 alleles included in $D_{\text{bot}}^{\left(\text{gen}\right)}$, defining this benchmark as $D_{C100}^{\left(\text{gen}\right)}$. As with $D_{\text{bot}}^{\left(\text{gen}\right)}$, we predicted binding cores using DeepMHCII and derived putative TCR motifs for conditional generation. To study allele transferability, i.e. to make partial peptides that originally bound one allele now bind another, we created $D_{\text{shuffle}}^{\left(\text{gen}\right)}$ by shuffling alleles within their respective groups (DR, DP, and DQ) in $D_{C100}^{\left(\text{gen}\right)}$. For classification evaluation, we subsampled artificial negatives from the CAPTAn dataset to achieve a balanced 1:1 positive: negative ratio, creating $D_{C1\text{:}1}^{\left(\text{cls}\right)}$.

### 2.3 Peptide display assay

To experimentally validate computationally generated peptides, we used a cell-based peptide display assay that reports peptide–MHC-II binding as retention of a surface epitope tag. Each construct (pLX307 backbone) encodes, from the N-terminus: a candidate peptide, a c-Myc epitope tag, a human rhinovirus (HRV) 3C protease cleavage site, and the target HLA class II heterodimer. Co-expression with a membrane-anchored HRV 3C protease (pcDNA3.1 backbone) cleaves the linker between the Myc tag and the HLA ectodomain. Peptides that bind stably in the HLA groove retain the Myc tag at the cell surface, whereas non-binding peptides lose the surface Myc signal.

For each of two HLA allele pairs, DQB106:03/DQA101:03 and DQA101:02/DQB105:02, we tested 23 peptides: 13 computationally generated, the original parental peptide, CLIP, four positive controls, and four negative controls. Codon-optimized oligonucleotides encoding each peptide were cloned into linearized parental vectors (BlpI/XmaI digestion followed by In-Fusion assembly; Takara) and verified by Sanger sequencing.

HEK293T cells were transiently co-transfected with individual peptide–HLA constructs and the 3C protease–GFP reporter using TransIT-LT1 (Mirus Bio). At 48 and 72 hours post-transfection, cells were stained with Alexa Fluor 647–conjugated anti-c-Myc (clone 9E10; BioLegend) and PE-conjugated anti-HLA-DQ (clone HLADQ1; BioLegend) antibodies and analyzed on a Cytek Aurora spectral flow cytometer. Successfully transfected cells were gated on GFP positivity, and peptide–HLA binding was quantified as the delta median fluorescence intensity (ΔMFI) of the Myc signal relative to unstained controls. All experiments were performed in three independent replicates.

### 2.4 Baseline methods

As baselines for our generative model we used a frequency based model, a GibbsCluster2.0 (Andreatta *et al*. 2017) based model, and a left-to-right autoregressive model (LR). The frequency based model consisted of amino acid frequencies calculated separately for each allele of its binding peptides (<500nM). Similarly, we took all positive peptides for each allele and clustered the peptides with GibbsCluster 2.0, retaining the amino acid frequencies of the binding cores and the flanking regions separately. Details of the Gibbs method can be found in Appendix Section 4, available as supplementary data at *Bioinformatics* online. The LR model used the same architecture and conditioning as PepGen. We also compared different variations of PepGen using different hyperparameters for $P_{\text{var}}$ and $P_{\text{single}}$. Furthermore, we ablated the use of negative data by training the model only on binding peptides and no affinity conditioning (pos), training on all peptides using continuous affinity conditioning (base method), and using all peptides but using binary conditioning based on high binders (log transform above 0.63; bin).

### 2.5 Evaluation metrics

The binding quality of generated peptides was quantified using DeepMHCII discriminator scores (You *et al*. 2022), trained independently on the held-out test data (see Section 2.2 and Appendix Section 9, available as supplementary data at *Bioinformatics* online for classifier validation). These continuous scores provide threshold-free estimates of binding affinity distribution. We report three primary metrics (all macro-averaged across conditionings/alleles):

- **Mean BA**: Average DeepMHCII score per conditioning/allele
- **Top-1k BA**: Mean of top 1000 scores per conditioning/allele, emphasizing strong binders and generation diversity
- **High-binder coverage**: Number of conditionings yielding ≥100 or ≥1000 peptides above thresholds (10,50,100,500 nM)

Discriminative performance benchmark used AUROC with 500 nM threshold for BA data and artificial negatives for EL data (see Section 2.2).

## 3 Results

### 3.1 Generation evaluation

**Evaluation on**  $D_{\text{bot}}^{\left(\text{gen}\right)}$**:** We first evaluated high-binder generation on the NetMHCIIpan-derived $D_{\text{bot}}^{\left(\text{gen}\right)}$ benchmark (see Table 1). The task was to generate 10k (10 368) binding peptides for the given allele and the partial peptide. All models used BA conditioning set to 1 where applicable (see column $c_{BA}$ in Table 1) and duplicate removal; PepGen and the LR baseline additionally used post-training scheduling, which is not applicable to frequency or Gibbs baselines. The use of negative data points (BA>500nM corresponding to approximately <0.43) is shown in column “Neg” in Table 1. All models were trained with 6 initializations.

**Table 1:** Benchmark results on $D_{\text{bot}}^{\left(\text{gen}\right)}$.^a^

Method	Neg	$P_{\text{single}}$	$P_{\text{var}}$	$c_{\text{BA}}$	Mean score	Top 1000
PepGen(pos)	×	1	1	×	$0.401\;\pm_{0.015}$	$0.563\;\pm_{0.016}$
PepGen(bin)	√	1	1	≈	$0.394\;\pm_{0.033}$	$0.539\;\pm_{0.034}$
PepGen(var)	√	0	1	√	$0.403\;\pm_{0.008}$	$0.5700\;\pm_{0.013}$
PepGen(single)	√	1	0	√	$0.427\;\pm_{0.023}$	$0.554\;\pm_{0.015}$
PepGen(half)	√	0.5	0.5	√	$0.435\;\pm_{0.020}$	$0.566\;\pm_{0.017}$
PepGen	√	1	1	√	$0.447\;\pm_{0.019}$	$0.579\;\pm_{0.013}$
PepGen (ELBA)	√	1	1	√	$0.447\;\pm_{0.021}$	$0.583\;\pm_{0.013}$
PepGen (C0G)	√	1	1	√	$0.457\;\pm_{0.020}$	$0.586\;\pm_{0.014}$
LR	√	NA	NA	√	$0.412\;\pm_{0.009}$	$0.549\;\pm_{0.013}$
Freq	×	NA	NA	×	$0.348\;\pm_{0.014}$	$0.529\;\pm_{0.021}$
Gibbs	×	NA	NA	×	$0.362\;\pm_{0.013}$	$0.547\;\pm_{0.020}$

a Macro-averaged scores (mean/mean top-1k) over allele-peptide conditionings of 10k generated sequences. The mean and standard deviation across 6 initializations is shown. Columns indicate: use of negatives in data (Neg), single and variable length masking probabilities ($p_{\text{single}}$ and $p_{\text{var}}$), and if BA conditioning was used in the model ($c_{\text{BA}}$); not used, scalar and binary conditioning (×, √, and ≈, respectively). Highest scores are shown in bold.

PepGen with scalar BA conditioning achieved the highest mean BA (0.4470 PepGen and 0.4474 PepGen (ELBA), which includes EL data in the train set). PepGen outperformed frequency sampling (0.3476), Gibbs clustering (0.3616), and left-to-right auto-regressive (LR; 0.4116) models, while surpassing the binding threshold (BA≈0.43). Scalar BA conditioning outperformed both positive-only training [PepGen (pos)] and binary conditioning [PepGen (bin) with thr. 0.63], likely because it leverages all affinity data without arbitrary thresholds. Among PepGen variants (compare: var, single, half and PepGen), complete but balanced masking ($P_{\text{single}}=1$, $P_{\text{var}}=1$, $P_{\text{span}\_\text{type}}=0.8$) performed best. Notably, the differences were not as large as compared to other methods, excluding the underperforming PepGen(var). To further test the utility of the scalar BA we tried class 0 guidance (C0G) where we substituted the unconditional logits, using *null* conditioining, with y=0 and the same MHC as for the conditional logits. For BA trained PepGen this gave a minor improvement whereas applied for ELBA trained PepGen and LR models this did not give any improvement (Table 1 and Appendix Table 6, available as supplementary data at *Bioinformatics* online).

To assess the models’ capabilities in an optimization task, i.e. generation of the strongest binders, we evaluated the mean BA of the top-1k sequences among the 10k generated samples. Because duplicates were removed, the top-1k statistic is more sensitive to the diversity of generations. PepGen and LR models yielded approximately 8k–9k unique peptides (Appendix Table 6, available as supplementary data at *Bioinformatics* online), reflecting the explicit quality–diversity control via scheduling for PepGen and LR. Top-1k rankings broadly mirrored the overall means, but with narrower gaps. PepGen (0.5788) again outperformed all baselines, e.g. LR model with mean top-1k 0.5487. EL augmentation gave only marginal improvements for both mean and top-1k BA over BA-only training. Overall, all scalar-BA PepGen variants except PepGen (var) outperformed the baselines on both mean and top-1k BA.

**Figure 2:**
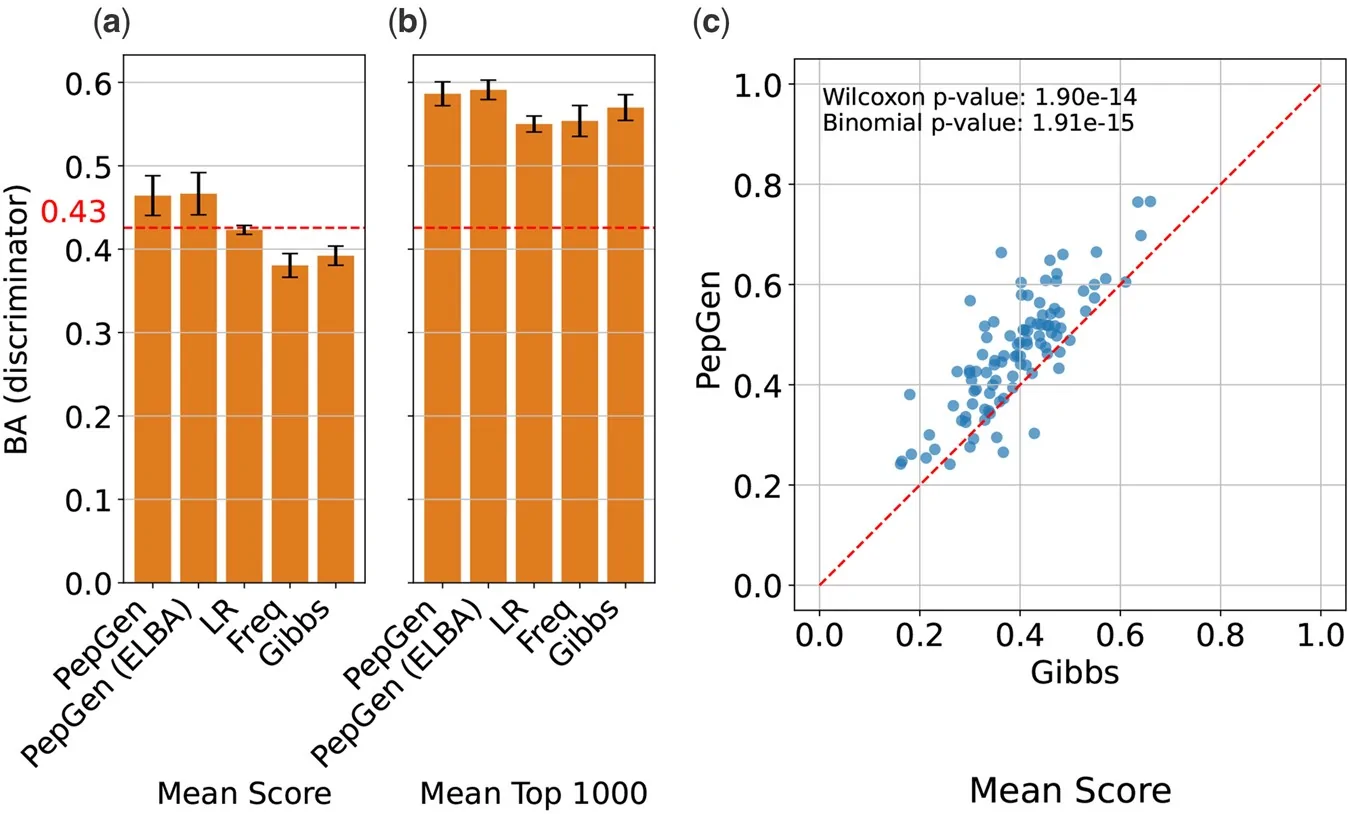
Generation performance on $D_{C100}^{\left(\text{gen}\right)}$. (a) Mean BA across unique sequences from 10k generations and (b) mean BA of top-1k unique sequences per conditioning. For each of 100 conditionings (allele + peptide motif + BA = 1), we generated 10k sequences, removed duplicates, computed per-conditioning mean and mean top-1k, then took the macro-average across conditionings. The bars show the mean macro-average and standard deviation across 6 initializations. The binding threshold of 500nM≈0.43 is marked in red. (c) Condition specific mean BA between PepGen and Gibbs on $D_{C100}^{\left(\text{gen}\right)}$. Each dot represents the mean score of one conditioning (allele, peptide conditioning and BA = 1) over 6 runs for Gibbs (*x*-axis) and PepGen (*y*-axis).

**Evaluation on**  $D_{C100}^{\left(\text{gen}\right)}$**:** We further benchmarked PepGen on $D_{C100}^{\left(\text{gen}\right)}$, where the conditioning partial peptides derive from original pMHC pairs with positive EL labels that imply at least modest binding affinity. Again, PepGen outperformed all baselines on both mean BA and top-1k BA (see Fig. 2a and b). Median BA values closely tracked means across models. Most compellingly, PepGen (63.5) and PepGen (ELBA) (62.3) achieved over 60 conditionings with median BA exceeding the binding threshold, compared to <50% coverage (48/100) for the LR baseline across the six initializations. Per-conditioning comparisons (Fig. 2c and Appendix, available as supplementary data at *Bioinformatics* online) confirmed PepGen’s advantage: it outperformed baselines on mean BA across most conditionings (despite Gibbs winning isolated cases), while maintaining significantly higher top-1k BA (P<.001) despite Gibbs showing competitive performance on some conditionings.

**Figure 3:**
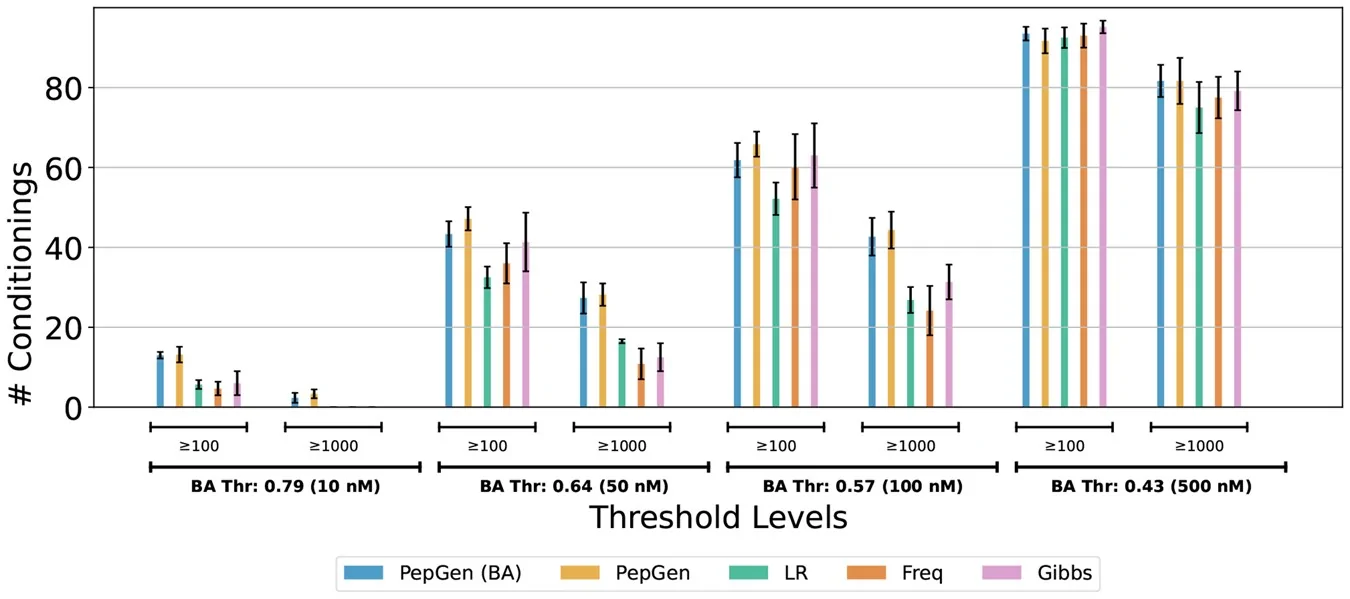
Number of conditionings (allele, partial peptide and y=1) that has more than 100 or more than 1000 binders over thresholds 10/50/100/500 nM. For each of the 100 conditionings 10k peptides were generated and duplicates removed. Evaluated on $D_{\text{shuffle}}^{\left(\text{gen}\right)}$. The bars show the mean and standard deviation across 6 initializations.

**Evaluation on**  $D_{\text{shuffle}}^{\left(\text{gen}\right)}$**:** We also evaluated performance using shuffled alleles within allele groups, which yielded very similar results to the original allele pairings but with slightly lower scores across all methods (Appendix Table 6, available as supplementary data at *Bioinformatics* online). Compared to the $D_{\text{bot}}^{\left(\text{gen}\right)}$ task, both $D_{C100}^{\left(\text{gen}\right)}$ and $D_{\text{shuffle}}^{\left(\text{gen}\right)}$ used positive-derived partial peptides, rendering these tasks easier overall, particularly benefiting frequency and Gibbs baselines, which handle partial conditioning less explicitly than PepGen. Finally, we quantified the number of conditionings (out of 100) yielding ≥100 or ≥1000 high binders at varying thresholds (Fig. 3). All methods generated ≥100 or ≥1000 binders for comparable numbers of conditionings at the 500 nM threshold (BA≈0.43). However, at stricter thresholds, especially requiring ≥1000 binders, PepGen consistently outperformed the baselines.

Binding cores are predicted by DeepMHCII and determine both the anchor positions that are generated and the conditioning subsets; BA values are likewise obtained from DeepMHCII. Evaluation scores could, therefore, in principle be inflated by exploiting its biases. We benchmark PepGen on conditionings independent of DeepMHCII, again showing superior performance over all baselines (Appendix Table 5, available as supplementary data at *Bioinformatics* online).

**Evaluation for *de novo* generation:** We also evaluated *de novo* generation with no partial peptide conditioning (Table 2). Using the same 37 alleles, we generated 10 368 peptides per method. This unconstrained task improved scores across most methods. PepGen again outperformed baselines, achieving mean BA of 0.5537 (BA-only training) and 0.5398 (EL-augmented). Both mean and top-1k performance exceeded baselines by ∼0.1units, demonstrating an increased performance margin as modeling freedom expands.

**Table 2:** Generation without peptide conditioning “*de novo*” for the same 37 alleles as in $D_{\text{bot}}^{\left(\text{gen}\right)}$.^a^

Method	Mean score	Top 1000	Num unique
PepGen BA	0.5537±0.0130	0.7160±0.0054	9080.2±167.7
LR	0.4058±0.0316	0.5977±0.0050	7529.4±1060.4
PepGen ELBA	0.5398±0.0272	0.7034±0.0127	9172.4±176.4
Freq	0.3971±0.0156	0.6016±0.0187	10368.0±0.0
Gibbs	0.4186±0.0105	0.6264±0.0113	10367.8±0.2

a A total of 10 368 peptides were generated per allele. The scores are mean macro-average over the allele scores (mean/mean top-1k) and standard deviation across 6 initializations. Highest scores are shown in bold.

### 3.2 Generative model as a discriminator

Here we demonstrate how the PepGen generative model can also be used as a classifier by utilizing sequence probabilities as classification scores (see Section 2.1.2 for details on classification score). This enables classification without negative training examples, which in theory has a key advantage for EL data where true non-binders are unavailable. Training and a meaningful comparison of discriminative models, however, require negatives in the train/test set.

**Figure 4:**
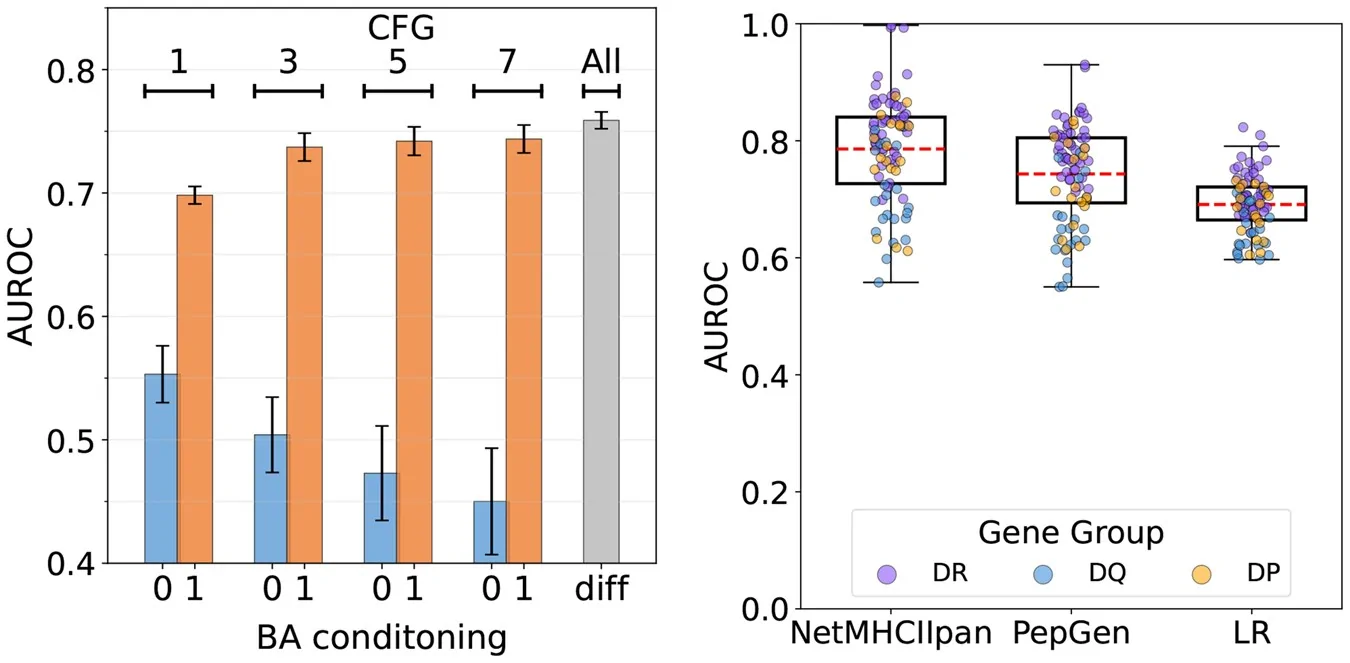
(left) Effect of CFG and BA conditioning values on classification. Sequence scores used for classification were evaluated with y=1 and y=0 on four CFG levels (*s*, shown above bars). The difference score (diff) is the same regardless of CFG weight. The bars show the mean and the standard deviation across 6 initializations. (right) Classification performance evaluation on $D_{C1\text{:}1}^{\left(\text{cls}\right)}$ against state of the art discriminative model NetMHCIIpan4.3.

We first examined BA conditioning and CFG effects on the test fold $D_{1-i,\text{val}}$ (Fig. 4, left). Classification with BA=1 improved monotonically with increasing CFG weight, confirming that stronger quality guidance enhances discriminative performance. Interestingly, the 0 BA conditioning starts at a level just above random with mean AUROC 0.55 when no CFG is used (s=1) and then decreases to 0.45 when s=7. Highest AUROC was achieved via the log-probability difference (or probability ratio) between BA=1 and BA=0 scores (see diff in Fig. 4 and Appendix Fig. 7, available as supplementary data at *Bioinformatics* online).

To compare against a state-of-the-art discriminative model (NetMHCIIpan4.3) we used the EL $D_{C1\text{:}1}^{\left(\text{cls}\right)}$ benchmark. As EL classification score cannot be evaluated as the difference between positive and negative scored we used the CFG weight s=7. While NetMHCIIpan4.3 performed better on most alleles PepGen performed remarkably well, given that it only used positively labeled datapoints for training, and was also better than the LR model (Fig. 4, right).

### 3.3 Experimental validation

To demonstrate the allele-switching capability of *PepGen*, we selected a SARS-CoV-2 peptide–TCR–pMHC complex (TEGALNTPKDHIGTR, HLA-DQA1*01:03-DQB1*06:03) from (Stražar *et al*. 2023). We aimed to redesign the peptide for an alternative allele from the same patient (HLA-DQA1*01:02-DQB1*05:02), which shares similar external residues but differs in binding groove amino acids, while preserving the putative TCR motif. We generated two peptide sets using PepGen conditioned on the alternative allele:

- **Free generation** (n=4): putative TCR motif fixed, remaining positions optimized; _____NT_K_HI___
- **Anchor-only** (n=9): only P1, P4, P6, P9 anchors varied from original peptide, testing whether anchor optimization alone suffices despite groove differences; TEGA_NT_K_HI_TR

Candidates were selected by *PepGen* classification score from a larger batch of generated peptides. Generated peptides, plus original peptide, CLIP control, and positive/negative controls (n=23), were tested by a cell-based display assay (Section 2.3)

**Figure 5:**
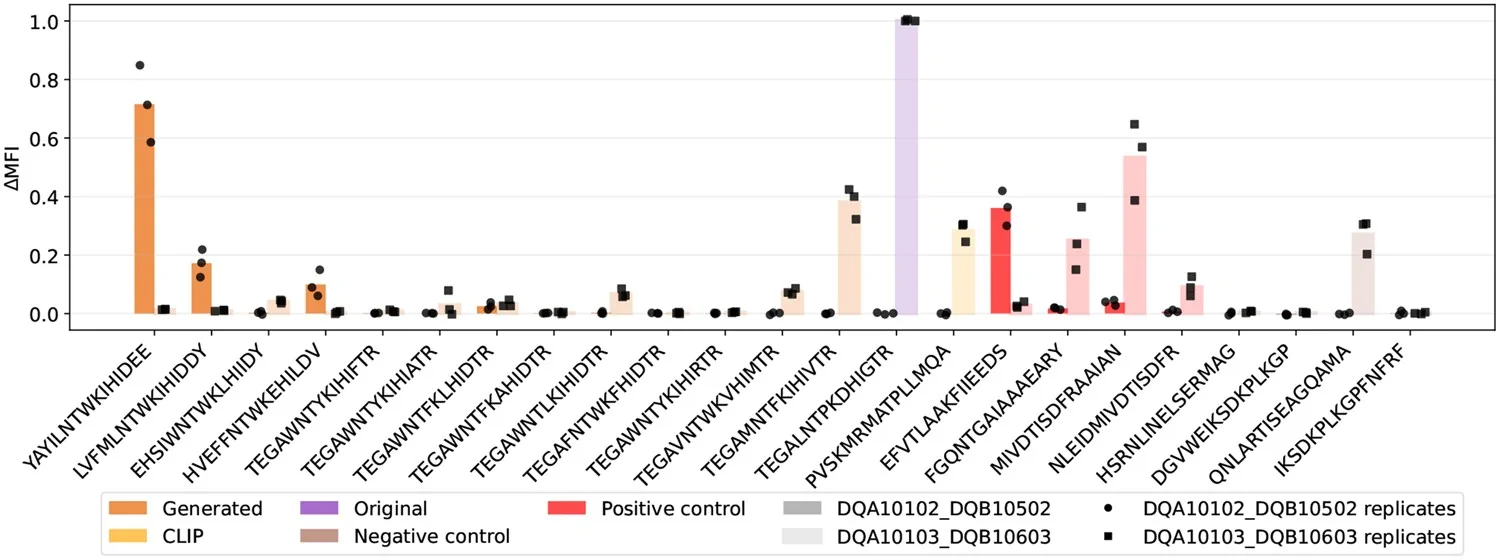
Results of the cell-based peptide display assay. HEK293 cells were co-transfected with a construct encoding the peptide, a c-Myc epitope tag, an HRV 3C protease cleavage site, and the target HLA-II heterodimer; peptides that stably bind the HLA-II heterodimer retain the Myc tag at the cell surface. Bars show mean ΔMFI across three replicates after c-Myc antibody staining, with individual replicates overlaid. We tested 13 PepGen-generated peptides alongside the original parent peptide, CLIP, four positive, and four negative controls; generated peptides are ordered by decreasing predicted binding score.

The original peptide did not bind the alternative allele, confirming allele-specific redesign was necessary. Three generated peptides bound: three of four from free generation (75%) and none of nine from anchor-only (Fig. 5). The original peptide to native allele showed highest normalized MFI (=1), while the best alternative allele binder reached approximately 0.7 relative MFI with the rest having less than 0.2. By construction, all three retained the target putative TCR motif, confirming *PepGen*’s ability to generate allele-compatible binders while enforcing motif preservation, with success dependent on positional freedom.

## 4 Discussion

PepGen addresses a critical gap in MHC II peptide generation by providing the first fully generative model capable of synthesizing peptide binders conditioned on allele, partial sequences, and continuous affinity. Its superior performance across diverse benchmarks stems from three key innovations: (i) addition of *single spans* with high-ratio GLM training emphasizing generative capabilities, (ii) scalar BA conditioning exploiting full affinity distributions, and (iii) post-training scheduling balancing quality-diversity tradeoffs.

PepGen consistently outperformed frequency, Gibbs, and autoregressive baselines, highlighting the advantage of bidirectional context modeling for MHC II. Notably, PepGen produced peptides with mean binding scores above threshold, with performance margins widening further in unconstrained *de novo* generation. These results demonstrate PepGen’s capacity to efficiently explore peptide space and generate high-affinity MHC II binders from partial peptides and from scratch.

Wet-lab validation provides compelling proof-of-concept for allele switching while preserving the putative TCR motif. Free generation yielded 3/4 binders versus none for anchor-only redesign, underscoring the critical role of non-anchor residues in MHC II binding. Notably, the original peptide failed to bind the alternative allele, while PepGen-generated variants achieved relative MFI up to 0.7. When assessed none of the three pMHC binders activated the TCR, consistent with the difficulty of peptide-TCR prediction even with correct MHC binding and motif preservation (Ishii *et al*. 2023). The putative TCR motif may also deviate from the true functional motif, and whether the alternative allele supports the same TCR remains unknown.

PepGen has a few limitations. First, our primary evaluation relies on DeepMHCII as a surrogate scoring model, which cannot fully substitute for experimental binding measurements at scale. Second, wet-lab validation is limited to a single TCR-pMHC system; large-scale validation is beyond the scope of this study. Third, MHC-II binding is necessary but not sufficient for T cell activation. While direct TCR-sequence conditioning remains challenging, motif-constrained infilling provides a feasible path toward dual MHC-TCR compatible peptides, though the motif must first be determined and validated. Future extensions could leverage emerging pMHC-TCR triplet datasets for joint conditioning or incorporate structural modeling to explicitly register core positions within the binding groove.

## Supplementary Material

btag464_Supplementary_Data

## Data Availability

Data is available via GitHub https://github.com/DaniTheOrange/PepGen. and Zenodo DOI: 10.5281/zenodo.21235044.

## References

[btag464-B1] Andreatta M , Alvarez B, Nielsen M et al GibbsCluster: unsupervised clustering and alignment of peptide sequences. Nucleic Acids Res 2017;45:W458–63. 28407089 10.1093/nar/gkx248 PMC5570237

[btag464-B2] Chen Z , Zhang B, Guo H et al Binding peptide generation for MHC class I proteins with deep reinforcement learning. Bioinformatics 2023;39:btad055. 10.1093/bioinformatics/btad055 PMC9907221 36692135

[btag464-B3] Dai Z , Huisman BD, Zeng H et al Machine learning optimization of peptides for presentation by class II MHCs. Bioinformatics 2021;37:3160–7. 33705522 10.1093/bioinformatics/btab131 PMC8504626

[btag464-B4] Du Z, Qian Y, Liu X et al *GLM: General Language Model Pretraining with Autoregressive Blank Infilling*. Proc. ACL, 2022.

[btag464-B5] Holtzman A, Buys J, Du L et al *The Curious Case of Neural Text Degeneration*. ICLR, 2020.

[btag464-B6] Ishii K , Davies JS, Sinkoe AL et al Multi-tiered approach to detect autoimmune cross-reactivity of therapeutic T cell receptors. Sci Adv 2023;9:eadg9845. 37494434 10.1126/sciadv.adg9845 PMC10371023

[btag464-B7] Nilsson JB , Kaabinejadian S, Yari H et al Accurate prediction of HLA class II antigen presentation across all loci using tailored data acquisition and refined machine learning. Sci Adv 2023;9:eadj6367. 38000035 10.1126/sciadv.adj6367 PMC10672173

[btag464-B8] Njirjak M , Žužić L, Babić M et al Reshaping the discovery of self-assembling peptides with generative AI guided by hybrid deep learning. Nat Mach Intell 2024;6:1487–500. ISSN 2522–5839.

[btag464-B9] Sanchez G, Spangher A, Fan H et al *Stay on Topic with Classifier-Free Guidance*. PMLR, 2024.

[btag464-B10] Stražar M , Park J, Abelin JG et al HLA-II immunopeptidome profiling and deep learning reveal features of antigenicity to inform antigen discovery. Immunity 2023;56:1681–98.e13. ISSN 1074–7613. 37301199 10.1016/j.immuni.2023.05.009 PMC10519123

[btag464-B11] Yang J, Chow IT, Sosinowski T et al Autoreactive T cells specific for insulin B: 11-23 recognize a low-affinity peptide register in human subjects with autoimmune diabetes. Proc Natl Acad Sci USA 2014;111:14840–5. 25267644 10.1073/pnas.1416864111 PMC4205657

[btag464-B12] You R, Qu W, Mamitsuka H et al DeepMHCII: a novel binding core-aware deep interaction model for accurate MHC-II peptide binding affinity prediction. Bioinformatics 2022;38:i220–8. 10.1093/bioinformatics/btac225 PMC9235502 35758790

