# Supplementary material for: PepGen: conditional generation of peptides for MHC binding
Source: Bioinformatics. 2026 Aug 21;42(Suppl 2):btag464. doi: 10.1093/bioinformatics/btag464 (PMC13501301; doi:10.1093/bioinformatics/btag464)
Supplement: Supplementary file 1 [file btag464_supplementary_data.pdf]

Appendix for PepGen: Conditional generation of peptides for MHC binding

Dani Korpela[1,2,*], Alexandru Dumitrescu[1,2], Martin Stražar[2,3], Rui Li[2], Ramnik J. Xavier[2], Daniel B. Graham[2], and Harri Lähdesmäki[1,*]

[1]Department of Computer Science, Aalto University, Espoo, Finland
[2]Broad Institute of MIT and Harvard, MA, USA
[3]Department of Biotechnology and Systems Biology, National Institute of Biology, Ljubljana, Slovenia
[*]Corresponding author. dani.korpela/harri.lahdesmaki @aalto.fi

May 15, 2026

1 Generation time parameter ablation

To determine suitable levels of classifier-free guidance (CFG) and nucleus sampling, we performed an ablation study across four settings for each parameter, [1, 0.7, 0.5, 0.3] for nucleus sampling and [1, 3, 5, 7] for CFG, resulting in 16 distinct combinations. Peptide sequences were generated based on six crystal structure–derived motifs. Anchor positions were manually annotated according to their spatial proximity and orientation toward the MHC molecule. The remaining residues within the binding groove, representing the peptide core, were assumed to form the TCR-interacting motif and were used as amino acid conditioning. For each of the six pMHC pairs, 5,120 peptides were generated for every CFG–nucleus sampling combination. For these sets, we evaluated the mean and top-100 discriminator scores along with the number of unique peptides (Appendix Fig. 1). Notably, substantial variation across all three metrics was observed between different pMHC pairs. Using a high CFG weight in combination with a low nucleus sampling value markedly reduced peptide diversity. In contrast, more balanced parameter settings yielded higher mean and top-100 scores as well as a greater number of unique peptides. Since the optimal configurations — maximizing both uniqueness and discriminator metrics — varied between conditioning contexts, we adopted a practical solution by implementing an adaptive scheduling strategy.

2 PepGen hyperparameters

We used a learning rate of 10^{-4}, a batch size of 64, $d_{\text{model}} = 1280$, 20 attention heads, $d_{\text{ff}} = 5120$, and a dropout rate of 0.1. The span length weights were set to $\lambda_{\text{single}} = 1$ and $\lambda_{\text{variable}} = 3$ for all model variants. The span-type probability $p_{\text{span\_type}}$ was set to 0.8, favoring single-span generation in all models except those restricted to a single span type (i.e., *PepGen (var)* and *PepGen (single)*).

3 Masking configuration probability

$$p(\mathbf{c}|L) = P_{\text{span,type}} \cdot P_{\text{var,len}} \cdot P_{\text{single,len}} \cdot P_{\text{last}} \cdot P_{\text{var,mask}} \\ \cdot P_{\text{single,mask}} \cdot P_{\text{order}}$$

The first term represents the probability of sampling N_{single} *single spans* and N_{var} *variable spans*, $P_{\text{span,type}} = p_{\text{span}}^{N_{\text{single}}}(1 - p_{\text{span}})^{N_{\text{var}}}$. The second and third terms represent the probabilities of sampling l length spans for *variable* and *single*, respectively,

$$P_{\text{var,len}} = \prod_{i \in \mathcal{G}} \text{Po}(l_i - 1|\lambda_{\text{var}})$$
$$P_{\text{single,len}} = \prod_{i \in \mathcal{U}} \text{Po}(l_i - 1|\lambda_{\text{single}}),$$

where $\mathcal{G}$ and $\mathcal{U}$ are *variable* and *single span* sets, excluding the last span (i.e., $|\mathcal{G}|+|\mathcal{U}|+1 = N_s = N_{single} + N_{var}$). As noted above, the span length sampling distributions for both *single* and *variable spans* are restricted by the length of the sequence $\mathbf{x}$, thus the span lengths are drawn from truncated Poisson distributions, where the remaining sequence length (not already attributed to some span) will have the probability mass of all exceeding lengths. This results in the following last span probability

$$P_{\text{last}} = 1 - \mathbb{1}[s_{N_s} = \text{var}] \cdot F_{\text{Po}(\lambda_{\text{var}})}(l_{N_s} - 2) \\ - \mathbb{1}[s_{N_s} = \text{single}] \cdot F_{\text{Po}(\lambda_{\text{single}})}(l_{N_s} - 2),$$

where we note that the Poissons are shifted by 1, and $F_{\text{Po}(\lambda)}$ is the Poisson CDF. The probabilities of masking *variable spans* $P_{\text{var,mask}} = p_{\text{var}}^{m}(1-p_{\text{var}})^{N_{\text{var}}-m}$ or tokens in *single spans* $P_{\text{single,mask}} = p_{\text{single}}^{b}(1-p_{\text{single}})^{\sum_{i=1}^{N_{single}} l_i - b}$ are both binomial, where m and b are the counts of masked spans and tokens in *single spans*, respectively. Lastly, $P_{\text{order}} = \frac{1}{(m+b)!}$ denotes the probability of the sampled generation order.

4 Gibbs generation method

Using core position acid frequencies $p_c(a_i|mhc)$ (for some allele mhc), sample amino acids independently according to

$$\mathbf{x} \sim \prod_{i=1}^{9} p(a_i|h) \tag{1}$$

For amino acids outside the core, we consider another distribution $p_o(a|h)$ (extensions of this method may consider $p_o(a_{\pm i}|h)$, for positions i outside the core).

We use GibbsCluster 2.0 to determine p_c and p_o. Concretely, both p_c and p_o are categorical distributions, with categorical likelihoods and dirichlet priors based on background frequencies, giving

$$p_c(a_i = j|h) = \frac{\alpha_j + c_{j,i}}{\sum_k \alpha_k + c_{k,i}}, \tag{2}$$

where α_k are determined by the background frequencies and a multiplicative prior factor f, and $c_{k,i}$ are the counts of each amino acid at each core position i (note that the prior is position-agnostic).

We create a similar setting, where some amino acids are known i.e., the partial peptide, and we want to "fill in gaps". The corresponding gaps are now fixed-length. To decide

the core position, we compute the average NLL for each known position, for each starting core position j

$$s_j = \sum_{i=1}^{9} \left(\mathbb{1}_{[i+j \in \mathcal{I}]} \log p_c(a_{i+j})\right)/C, \tag{3}$$

where C is the count of pre-conditioning amino acids found from position j to $j+9$

$$C = \sum_{i=1}^{9} \mathbb{1}_{[i+j \in \mathcal{I}]} \tag{4}$$

We then sample the core position according to Softmax($\mathbf{s}$), and then sample all missing amino acids according to p_c and p_o.

5 Experiment on randomly generated core conditionings

To evaluate generation performance on conditions entirely independent of DeepMHCII scoring and the amino acid distribution of real binding peptides, we generated random conditioning sequences. Specifically, we took the alleles and peptide lengths from $\mathcal{D}^{(\text{gen})}_{\text{C100}}$, sampled a core position for each peptide, and filled all non-anchor core positions with amino acids drawn from a uniform distribution, using allele-group-specific canonical anchor positions. We refer to this task as $\mathcal{D}^{(\text{gen})}_{\text{rand}}$. PepGen outperformed all baselines on this task as well and achieved mean performance above the 500nM binding threshold (Appendix Table 5).

6 Supplementary tables

Table 1: results on $\mathcal{D}^{(\text{gen})}_{\text{C100}}$

Method	Mean Score	Top 1000	Num Unique	Median
PepGen	0.4642 ± 0.0238	0.5863 ± 0.0143	8695.6 ± 166.1	$0.466 \pm_{0.025}$
PepGen (ELBA)	0.4665 ± 0.0253	0.5910 ± 0.0116	8625.4 ± 185.7	$0.469 \pm_{0.026}$
PepGen (no sched)	0.3935 ± 0.0131	0.5608 ± 0.0082	10493.2 ± 1.5	
PepGen (ELBA, no sched)	0.4087 ± 0.0091	0.5701 ± 0.0083	10486.2 ± 7.3	
LR	0.4231 ± 0.0053	0.5500 ± 0.0096	8115.3 ± 870.4	$0.424 \pm_{0.004}$
Freq	0.3805 ± 0.0142	0.5537 ± 0.0185	10367.9 ± 0.1	$0.380 \pm_{0.016}$
Gibbs	0.3922 ± 0.0115	0.5698 ± 0.0154	10345.9 ± 14.5	$0.392 \pm_{0.013}$
PepGen (0g)	0.4747 ± 0.0243	0.5946 ± 0.0152	8441.1 ± 146.5	$0.478 \pm_{0.026}$
PepGen (ELBA;0g)	0.4656 ± 0.0239	0.5890 ± 0.0128	8447.4 ± 194.8	$0.468 \pm_{0.025}$
LR (0g)	0.4171 ± 0.0025	0.5433 ± 0.0074	7957.8 ± 838.6	$0.417 \pm_{0.002}$

Table 2: Results on $\mathcal{D}^{(\text{gen})}_{\text{shuffle}}$

Method	Mean Score	Top 1000	Num Unique
PepGen	0.4571 ± 0.0224	0.5811 ± 0.0145	8681.8 ± 184.3
PepGen (ELBA)	0.4603 ± 0.0250	0.5875 ± 0.0119	8631.4 ± 162.2
LR	0.3886 ± 0.0106	0.5095 ± 0.0224	5380.9 ± 845.2
Freq	0.3753 ± 0.0165	0.5474 ± 0.0212	10367.9 ± 0.0
Gibbs	0.3864 ± 0.0137	0.5624 ± 0.0183	10308.0 ± 19.3
PepGen (0g)	0.4669 ± 0.0242	0.5889 ± 0.0174	8436.2 ± 153.7
PepGen (ELBA;0g)	0.4593 ± 0.0238	0.5853 ± 0.0136	8461.8 ± 195.0
LR (0g)	0.4127 ± 0.0043	0.5379 ± 0.0089	7960.5 ± 833.8

Table 3: Benchmark results on $\mathcal{D}^{(\text{gen})}_{\text{bot}}$

Method	Neg	$p(m_1)$	$p(m_{\text{var}})$	c_{BA}	Mean Score	Top 1000	Num Unique
PepGen	✓	0.8	1	✓	0.4470 ± 0.0187	0.5788 ± 0.0127	8564.3 ± 167.9
PepGen(pos)	×	0.8	1	×	0.4012 ± 0.0148	0.5632 ± 0.0164	9533.6 ± 241.5
LR	✓	NA	NA	✓	0.4116 ± 0.0086	0.5487 ± 0.0130	8279.3 ± 778.2
PepGen(bin)	✓	1	1	✓	0.3944 ± 0.0333	0.5386 ± 0.0337	8982.3 ± 454.2
PepGen (ELBA)	✓	1	1	✓	0.4474 ± 0.0214	0.5825 ± 0.0128	8487.4 ± 161.2
PepGen(var)	✓	0.0	1.0	✓	0.4030 ± 0.0083	0.5700 ± 0.0127	8261.3 ± 186.1
PepGen(single)	✓	1.0	0.0	✓	0.4266 ± 0.0233	0.5544 ± 0.0150	8289.8 ± 365.5
PepGen(half)	✓	0.5	0.5	✓	0.4346 ± 0.0199	0.5662 ± 0.0173	8770.0 ± 369.2
Freq	×	NA	NA	×	0.3476 ± 0.0142	0.5292 ± 0.0213	10518.2 ± 2.2
Gibbs	×	NA	NA	×	0.3616 ± 0.0127	0.5474 ± 0.0195	10491.8 ± 5.4
PepGen (0g)	✓	1	1	✓	0.4568 ± 0.0195	0.5863 ± 0.0143	8321.5 ± 177.6
LR (0g)	✓	NA	NA	✓	0.4055 ± 0.0055	0.5410 ± 0.0092	8093.8 ± 767.9
PepGen (ELBA;0g)	✓	1	1	✓	0.4456 ± 0.0196	0.5799 ± 0.0125	8290.8 ± 161.9

Table 4: *De novo*

Method	Mean Score	Top 1000	Num Unique
PepGen BA	$\mathbf{0.5537 \pm 0.0130}$	$\mathbf{0.7160 \pm 0.0054}$	9080.2 ± 167.7
LR	0.4058 ± 0.0316	0.5977 ± 0.0050	7529.4 ± 1060.4
PepGen ELBA	$\mathbf{0.5398 \pm 0.0272}$	$\mathbf{0.7034 \pm 0.0127}$	9172.4 ± 176.4
Freq	0.3971 ± 0.0156	0.6016 ± 0.0187	10368.0 ± 0.0
Gibbs	0.4186 ± 0.0105	0.6264 ± 0.0113	10367.8 ± 0.2
PepGen (0g)	0.5555 ± 0.0166	0.7134 ± 0.0081	9081.7 ± 144.4
PepGen (ELBA;0g)	0.5251 ± 0.0347	0.6925 ± 0.0208	9110.1 ± 199.7
LR (0g)	0.3713 ± 0.0418	0.5758 ± 0.0051	7515.3 ± 930.9
Freq	0.3971 ± 0.0156	0.6016 ± 0.0187	10368.0 ± 0.0
Gibbs	0.4186 ± 0.0105	0.6264 ± 0.0113	10367.8 ± 0.2

Table 5: $\mathcal{D}^{(\text{gen})}_{\text{rand}}$

Method	Mean Score	Top 1000	Num Unique
PepGen	0.4301 ± 0.0245	0.5556 ± 0.0184	8750.3 ± 143.7
LR	0.4029 ± 0.0104	0.5300 ± 0.0142	8340.9 ± 733.6
Freq	0.3554 ± 0.0160	0.5295 ± 0.0216	10367.9 ± 0.0
Gibbs	0.3730 ± 0.0140	0.5496 ± 0.0196	10337.6 ± 23.2

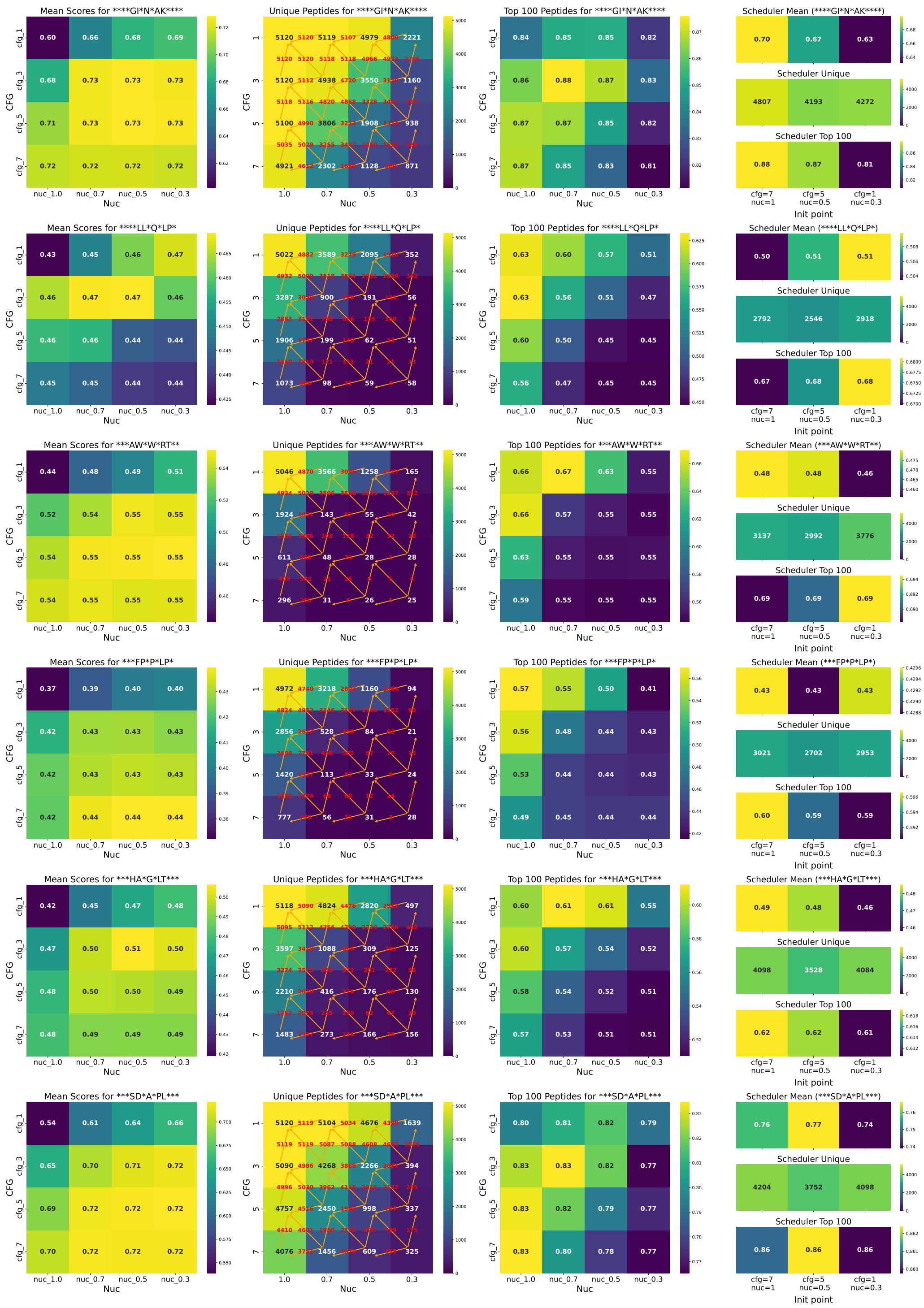

Figure 1: Generation time parameter ablation. Peptides were generated based on six pMHC pairs from crystal structures (rows). The columns show the mean score, the number of unique peptides and the top-100 score for the different values of CFG and nucleus sampling parameters; the last column shows the same scores using the adaptive scheduler from three initialization points. The red arrows show the number of new unique peptides from less diverse to more diverse parameters. All values are means of 6 initializations.

7 Supplementary figures

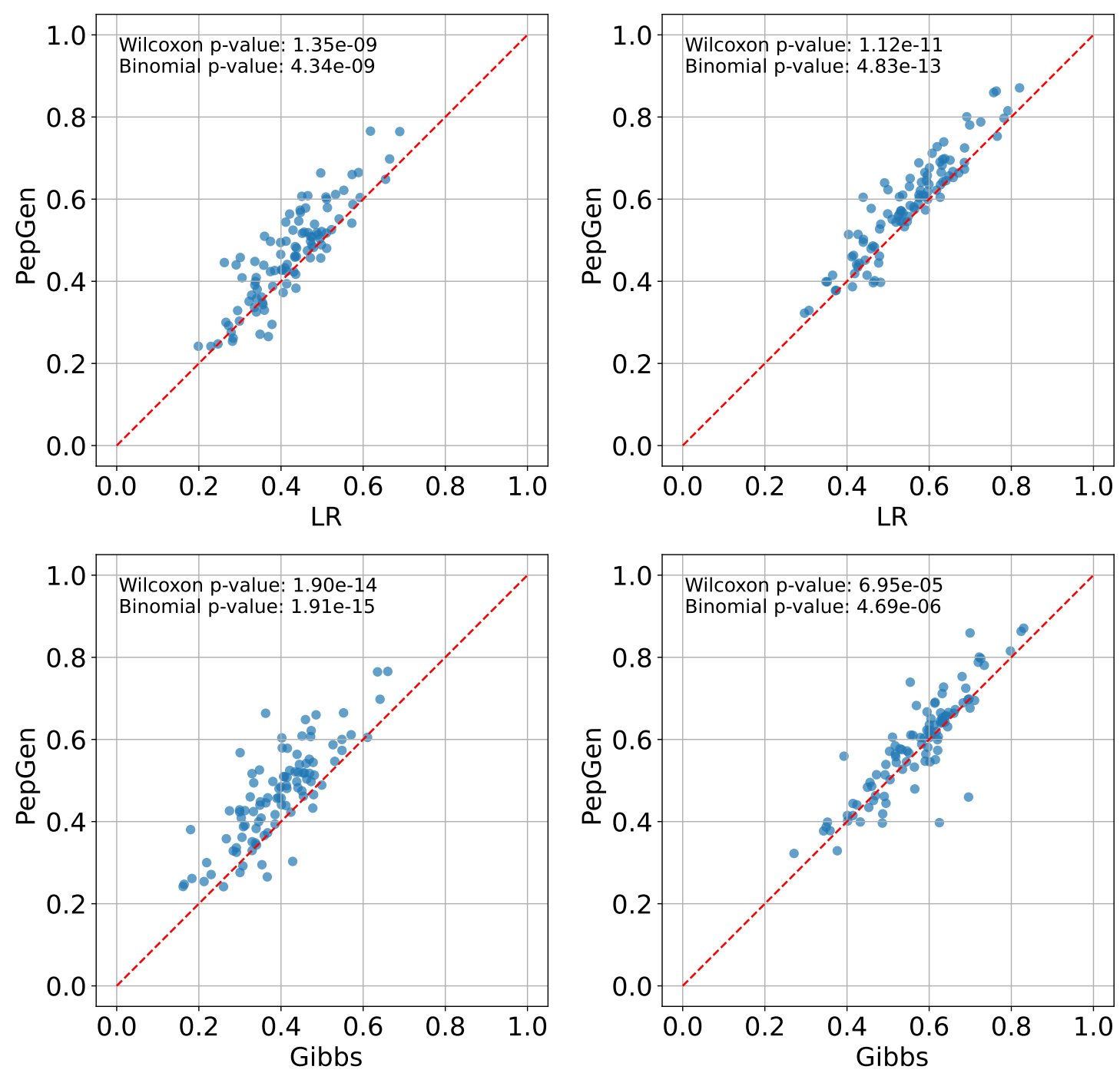

Figure 2: Condition specific mean BA (left) and top-1k (right) between PepGen and LR (upper) and Gibbs (bottom) on $\mathcal{D}^{(\text{gen})}_{\text{C100}}$. Each dot represents the mean score of one conditioning (allele, peptide conditioning and BA=1) over 6 runs for LR/Gibbs (x-axis) and PepGen (y-axis).

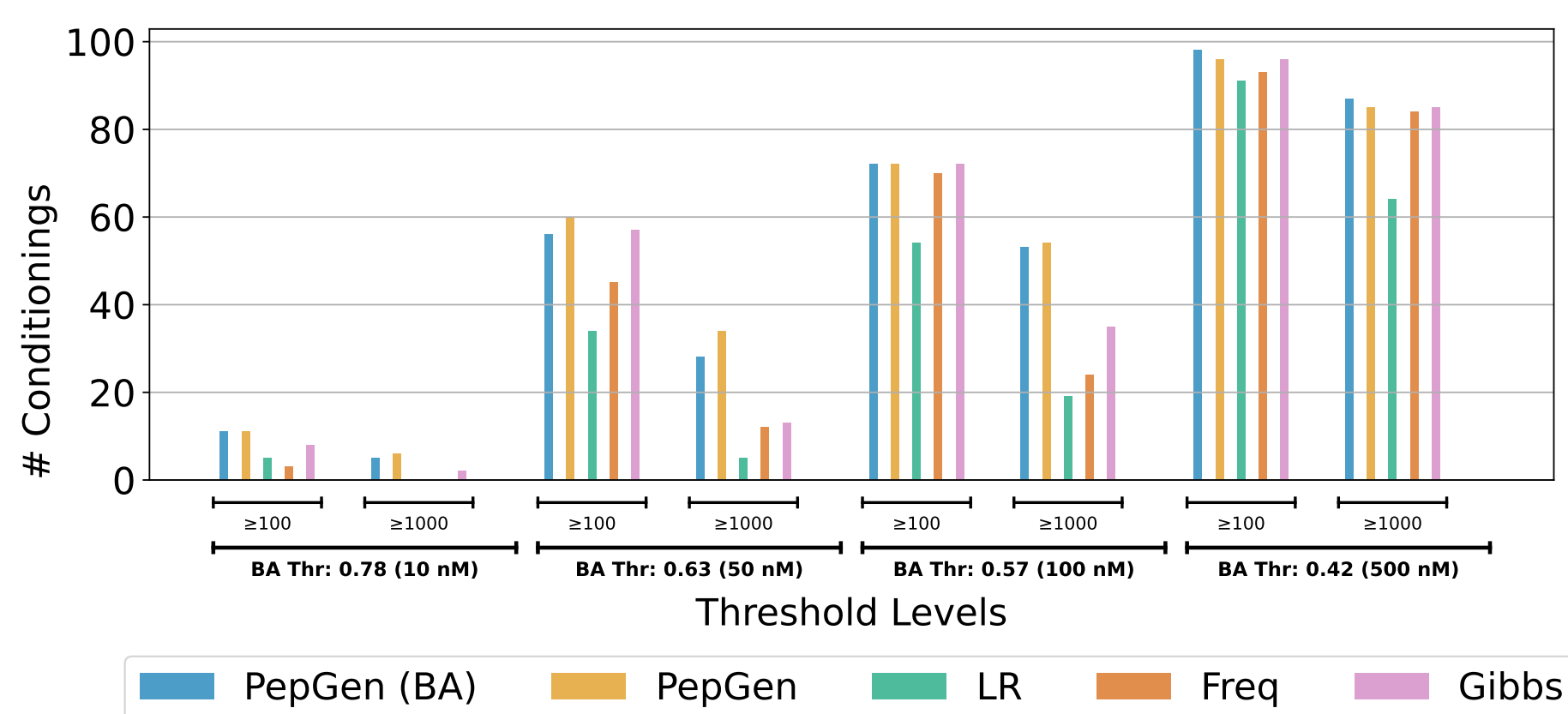

Figure 3: Number of conditionings (allele, partial peptide and $y = 1$) that has more than 100 or more than 1000 binders over thresholds 10/50/100/500nM. For each of the 100 conditionings 10k peptides were generated and duplicates removed. Evaluated on $\mathcal{D}^{(\text{gen})}_{\text{C100}}$. The bars show the mean and standard deviation across 6 initializations.

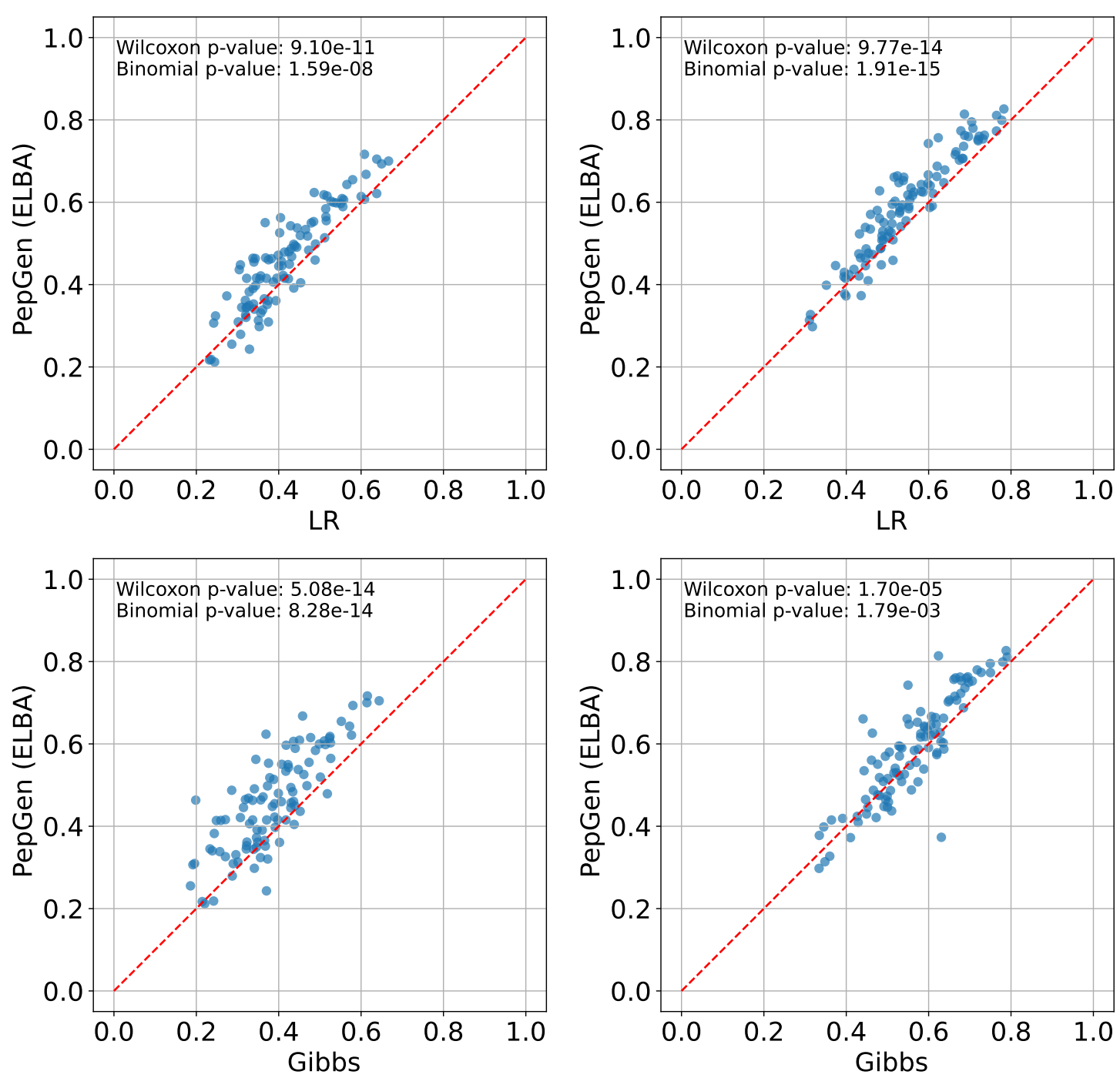

Figure 4: Condition specific mean BA (left) and top-1k (right) between PepGen and LR (upper) and Gibbs (bottom) on $\mathcal{D}^{(\text{gen})}_{\text{shuffle}}$. Each dot represents the mean score of one conditioning (allele, peptide conditioning and BA=1) over 6 runs for LR/Gibbs (x-axis) and PepGen (y-axis).

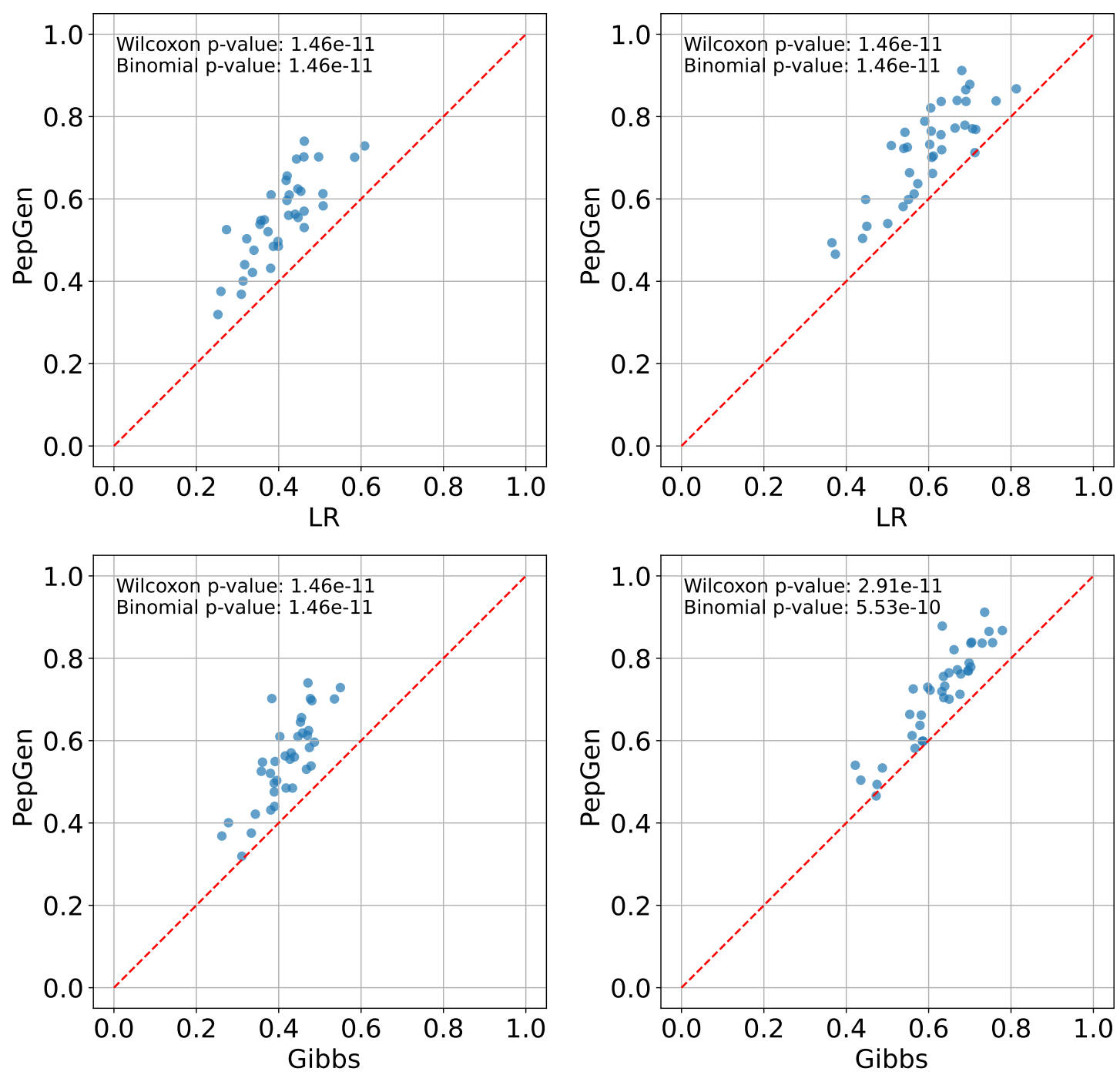

Figure 5: Allele specific mean BA (left) and top-1k (right) between PepGen and LR (upper) and Gibbs (bottom) on *de novo* generation. Each dot represents the mean score of one allele over 6 runs for LR/Gibbs (x-axis) and PepGen (y-axis).

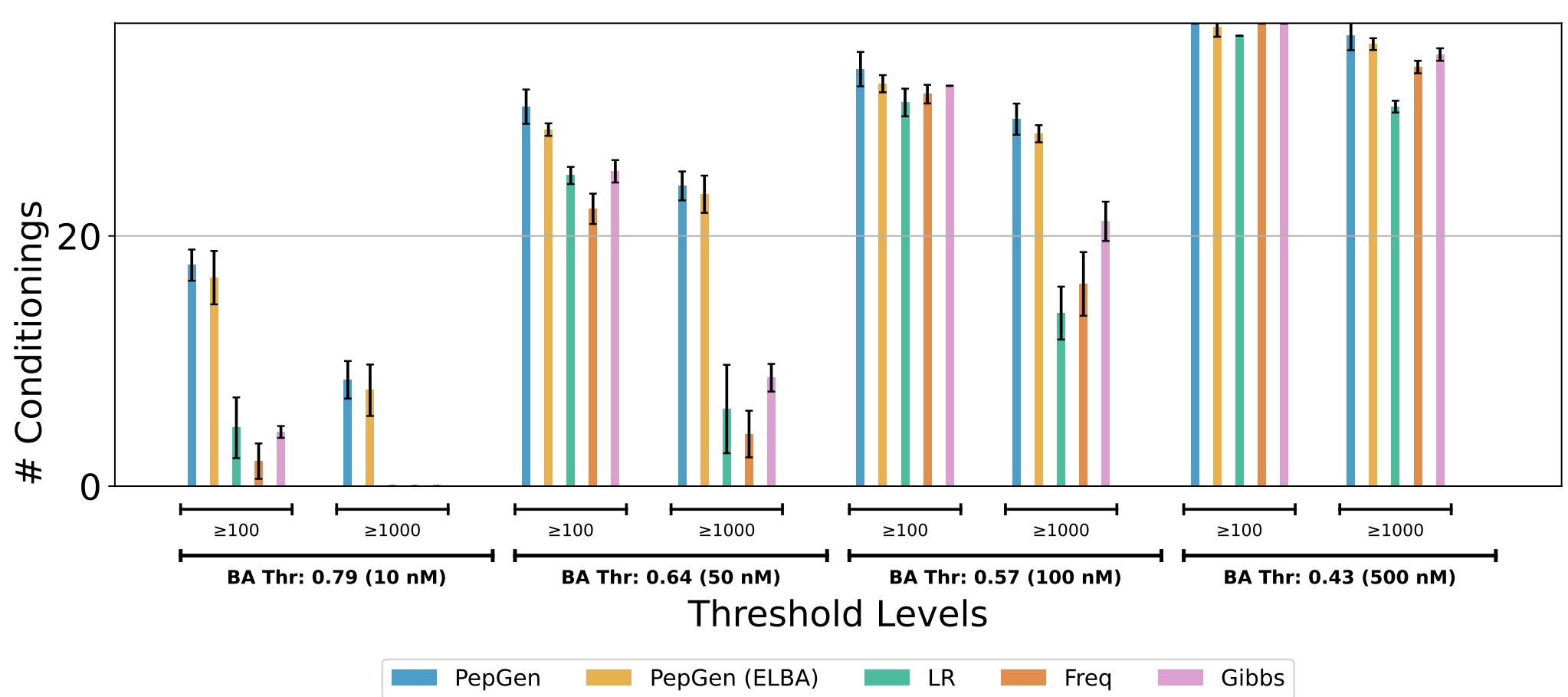

Figure 6: Number of alleles that has more than 100 or more than 1000 binders over thresholds 10/50/100/500nM on *de novo* generation. For each allele 10k peptides were generated and duplicates removed. The bars show the mean and standard deviation across 6 initializations.

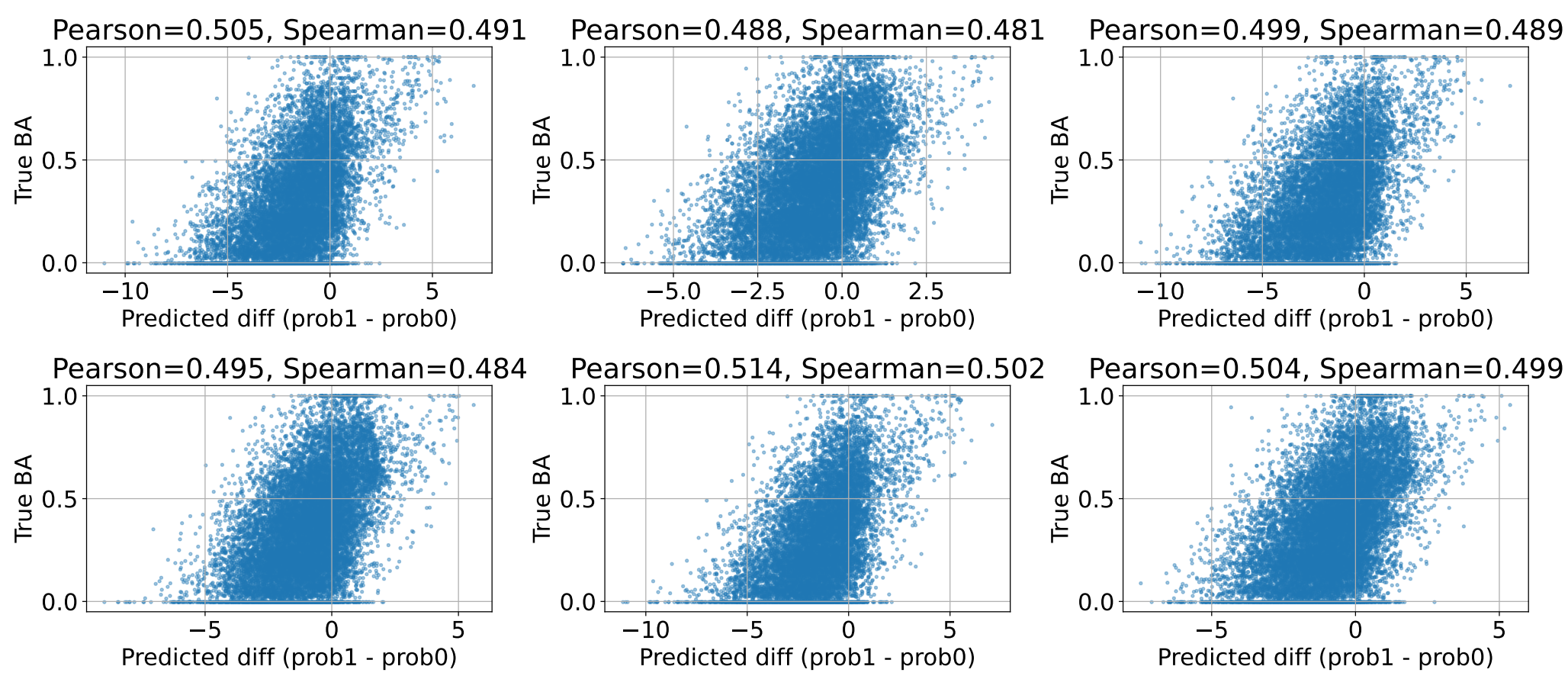

Figure 7: Scatterplot and correlation coefficients for the PepGen Diff classification score w.r.t. experimental binding affinity (BA) values for the 6 instances

8 Pseudocode for span configuration

Algorithm 1 GLM Masking Procedure for Peptide Sequence $\mathbf{x}$

Require: Peptide $\mathbf{x} = (x_1, \ldots, x_L)$, parameters $\{p_{\text{span}}, \lambda_{\text{var}}, \lambda_{\text{single}}, p_{\text{var}}, p_{\text{single}}\}$

Ensure: Masked input with span reconstruction targets

```
Part A ← ∅
masked_spans ← ∅
target_spans ← ∅
pos ← 1
while pos ≤ L do
    s ← Bernoulli(p_span)                          ▷ var=0, single=1
    λ ← s · λ_var + (1 − s) · λ_single
    l ← Poisson(λ) + 1
    end ← min(pos + l − 1, L)
    l ← end − pos + 1
    if s = 0 then                                  ▷ Variable span
        if Bernoulli(p_var) = 1 then
            Append m_v to Part A
            Append [S_v, x_[pos:end]] to masked_spans
            Append [x_[pos:end], e_v] to target_spans
        else
            Append x_[pos:end] to Part A
        end if
    else                                           ▷ Single-token span
        for i = pos to end do
            if Bernoulli(p_single) = 1 then
                Append m_s to Part A
                Append [s_s, x_i] to masked_spans
                Append [x_i, ∅] to target_spans
            else
                Append x_i to Part A
            end if
        end for
    end if
    pos ← end + 1
end while
Sample random permutation π of {1, ..., N_masks}
masked_spans ← (masked_spans_π(1), ..., masked_spans_π(N_masks))
target_spans ← (target_spans_π(1), ..., target_spans_π(N_masks))
Part B ← concat(masked_spans)
return Part A + Part B, concat(target_spans)
```

9 Classifier validation

To assess the performance of the DeepMHCII classifiers used as test oracles, we evaluated each classifier on the validation subset of the held-out fold: a classifier trained on $\mathcal{D}_i$ was evaluated on $\mathcal{D}_{1-i,\mathrm{val}}$. We report the Pearson correlation between the true and predicted binding values for each classifier.The two classifiers achieved Pearson correlations of $r = 0.71$ and $r = 0.68$, indicating strong predictive performance and good utility as test oracles (See 8).

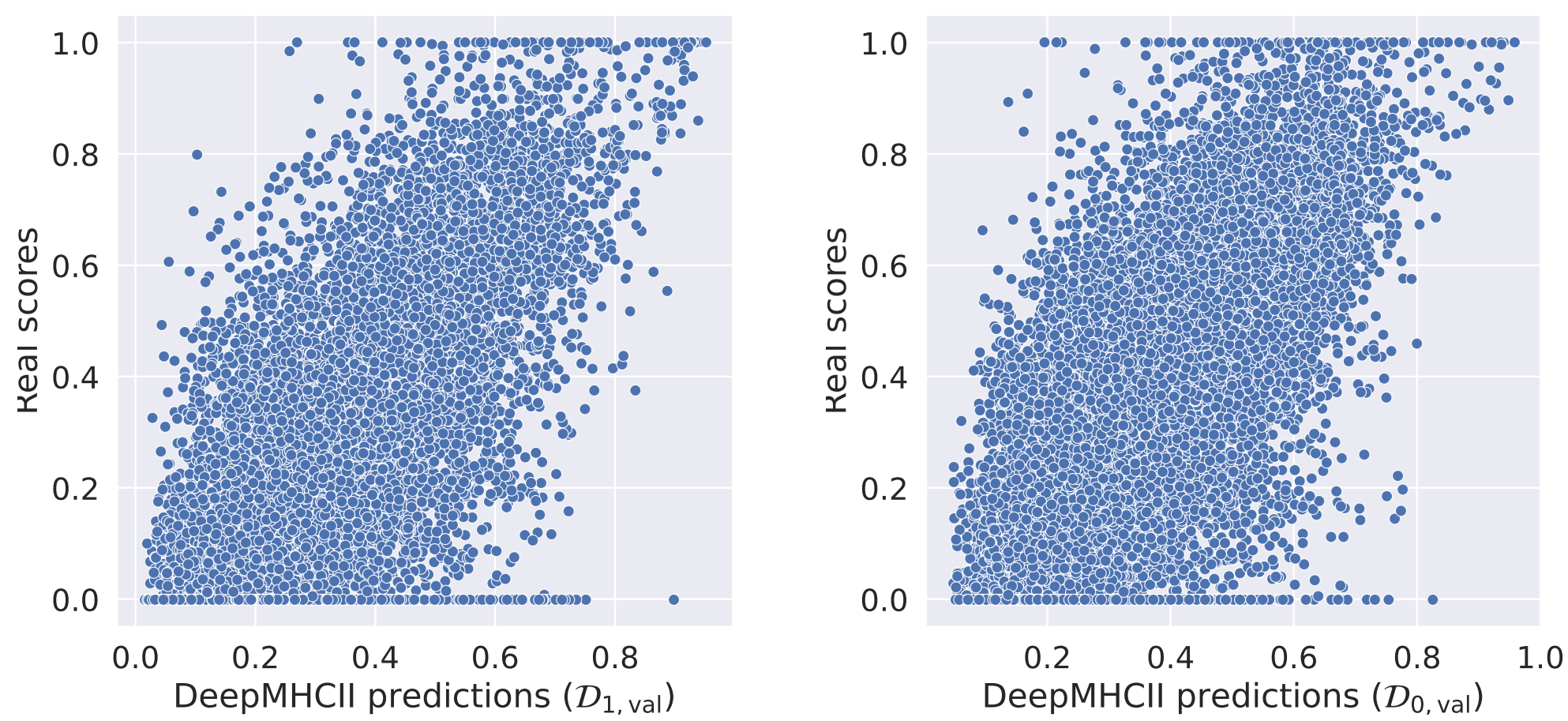

Figure 8: Scatterplots of experimental values against oracle predicted values for classifiers trained on D_0 (left) and $\mathcal{D}_1$ (right).
